# The magic of first impressions: Do facial displays on online platforms affect users’ offline conversion rate?

**DOI:** 10.1371/journal.pone.0347346

**Published:** 2026-06-03

**Authors:** Xue Zhang, Qiutong Li, Chunjia Han, Xinyue Huang

**Affiliations:** 1 School of economics and management, Harbin Huade University, Harbin, P.R. China; 2 Business school, Birkbeck, University of London, Bloomsbury, London, United Kingdom; 3 School of management, Heilongjiang University of Science and Technology, Harbin, P.R. China; Dong-A University College of Business Administration, KOREA, REPUBLIC OF

## Abstract

With the rise of Internet health, online medical platforms play the key role of information bridge and convenient channel in medical service selection. Users tend to rely on doctor images and electronic word-of-mouth evaluations displayed on online medical platforms when selecting medical services. This study aims to explore the impact of facial features displayed on online platforms on users’ offline conversion rate. It will help us understand the psychological mechanism behind users’ decisions from the perspective of the first impression effect. Python was used to collect pictures and information of 7547 doctors from “haodf.com”, a well-known online medical platform in China. Then, we used commercial software to calculate their facial feature values. The results showed that: Appearance attractiveness, smile, and gender of facial displays on online platforms have a significant positive relationship on users’ offline conversion rate. Satisfaction evaluations do not significantly regulate the relationship between appearance attractiveness and smile on users’ offline conversion rate, but have a significant negative moderating effect on the effect of gender. This study emphasizes the importance of the eWOM platform in image management. It also provides a new perspective for optimizing resource allocation and improving service efficiency. It has important theoretical and practical value for further promoting the innovation of online service systems and realizing the seamless connection between online and offline.

## 1 Introduction

In recent years, with the rapid development of Internet technology and the acceleration of digital transformation, online medical platforms have become an indispensable part of contemporary medical services. These platforms provide users a full range of multi-mode services such as online booking, remote consultation, and health information [1]. With its convenience and efficiency, online medical platforms have attracted a large number of users to give priority to online medical services when they have consultation needs. This trend not only brings users a convenient channel to avoid time and space constraints, but also helps to alleviate offline medical congestion and improve the rationality of medical resource allocation [2]. More importantly, when these users have actual medical needs, they are highly likely to choose the doctors they have consulted online before for offline diagnosis and treatment [3]. Considering that the conversion rate from online to offline is critical for both medical platforms and doctors, it’s worth exploring in depth what factors affect users’ offline conversion rate.

On online medical platforms, users tend to rely on different types of information disclosed by the platform, such as physician personal data, physician image, and evaluation to make an overall judgment of the doctor. Among them, images’ presence and attractiveness are factors that users cannot ignore when browsing online medical platforms [4]. Compared with text description, image information has advantages in arousing users’ interest and attracting attention [5]. Furthermore, the facial features in images and the resulting first impression are essential to the user’s selection behavior process. Facial features refer to the distinctive characteristics of an individual’s face, including attributes such as eyes, nose, and mouth, as well as factors such as facial attractiveness and trustworthiness [6]. Research in recent years has shown that facial features play an important role in many situations. For example, different facial features will have different effects on career choice, social interaction, and wage income [7]. People often form their first impressions of others through faces, which can influence subsequent judgments and decisions [8]. The first-impression effect—a psychological mechanism that shapes user behavior based on initial visual cues—is particularly salient in high-stakes decision contexts like healthcare. This aligns with the concept of thin-slicing in behavioral science, where individuals make rapid judgments using minimal information [9], and with findings in clinical settings where physicians’ visual cues significantly affect patient compliance and perceived competence [10]. In the online medical field, the doctor’s positive first impression can quickly build the user’s trust, laying a good foundation for subsequent consultations and treatment. For example, a doctor’s professional white coat can convey a professional and rigorous attitude, and a doctor’s friendly smile can make patients feel cared and respected [4,10]. Therefore, we have reasons to believe that some facial features displayed on online medical platforms will also have an important impact on users’ offline choice of doctors. In the field of online medical, most scholars have conducted studies centered around platform health information [2], user feedback [11], and doctor-patient interaction [12] through interviews or questionnaires. Previous studies have identified many factors that affect online medical platform users’ decision-making, such as physician voice characteristics [11], patient ratings [13], and online reviews [12]. Moreover, scholars have also widely discussed the important influence of image in the fields of tourism [5], social media [14], and micro-communities [15]. However, fewer studies have focused on how “exposure to” real images influences users’ online and offline behavior. We adopt the research method of visual image analysis, which is different from most studies based on review text analysis techniques. Therefore, the main purpose of this study is to explore how facial displays on online platforms affect users’ offline conversion rate from the perspective of the psychological first impression effect theory.

The impact of eWOM on consumer responses (e.g., trust and loyalty) has been extensively studied. Users have a strong reliance on positive eWOM on both social media and e-commerce platforms [16]. In the process of online medical services, user satisfaction evaluation is the user’s opinion and satisfaction level of the doctor’s service quality, which can be regarded as the doctor’s eWOM [17]. Most previous studies took “satisfaction” as an independent variable to study its impact on consumer decision-making [18], or took “satisfaction” as the dependent variable to explore its influencing factors and mechanisms [11]. However, satisfaction evaluation as a moderating variable is rarely studied. This study mainly includes efficacy and attitude satisfaction evaluation, which rates doctors’ professional skills and service attitude. eWOM expressed in terms of “satisfaction evaluation” will not only have a direct impact on user decision-making, but may also moderate the influence of certain attributes of the physician on user behavior, such as the physician’s facial image [4]. Therefore, this study also aims to explore how the online satisfaction evaluation moderates the impact of facial features on users’ offline consultation conversion rate.

In this study, we select facial features including appearance attractiveness, smile, and gender as explanatory variables. The reasons for choosing these three facial features are as follows. People form different impressions of faces mainly from two dimensions of profile pictures: changeable aspects and invariant aspects [19]. Changeable aspects represent the internal changes of people. It mainly includes emotion expression and viewpoint [4]. Among these facial indicators, appearance attractiveness is related to the viewpoint, and the smile is related to emotion expression [4]. Gender belongs to invariant aspects [19]. All three facial features may influence the user’s initial impression of the doctor. Scholars have proved that appearance attractiveness plays an important role in impression management. With the development and maturity of image recognition technology, the quantification and measurement of appearance are more accurate and reliable. Smiling is seen as friendly and trustworthy, and can convey positive feelings to others through “emotional contagion” [20]. Gender attraction and bias can also give people different impressions and affect people’s behavioral decisions. In addition, while there are many other indicators of facial features, appearance attractiveness, smile, and gender are the ones people pay more attention to, not to mention they are also the core components of facial contour. Therefore, we choose these three facial feature variables to solve the following research questions:

RQ1. How do online doctor image affect users’ offline conversion rate?
RQ2. How do satisfaction evaluations as eWOM regulate the relationship between online doctor image and users’ offline conversion rate?
RQ3. What are the differences in the impact of facial features on users’ offline conversion rate among doctors with different recommendation degrees?

In order to solve the above research questions, we collected data from the well-known domestic online medical platform haodf.com. The algorithm Face++ provided by MegVII Technology Co., Ltd. is used to calculate and analyze the attributes of doctor images. We utilize a commercial and well-known facial recognition service, Face++ (https://www.faceplusplus.com/), in our image analysis. That’s because Face++ represents the most advanced facial recognition technology and is widely used in academic research [21], such as analyzing social media profile photos [22]. Therefore, we used scientific and accurate Face++ facial recognition technology in this study to analyze the facial features of doctors’ images displayed on online medical platforms.

In this study, we have some important findings. The “beauty premium” phenomenon prevalent in the labor market also exists on online medical platforms. There is evidence to support that appearance attractiveness has a significant positive impact on users’ offline conversation rate. In addition, the satisfaction evaluation as an electronic word-of-mouth for physicians weakens the effect of gender on users’ offline conversion rate. This suggests that gender bias is also reduced after exposure to real healthcare satisfaction feedback. This study enriches the literature related to facial displays and eWOM on online medical platforms [23]. Previous studies have shown that users benefit from the information and emotions provided by the platform [24]. In this study, we first use visual image technology to quantify facial features. Subsequently, we explore the effect of facial displays on online platforms on user behavior from a psychological perspective, thus advancing the literature. This study addresses a significant gap by focusing on visual cues on online medical platforms and examining the impact of facial displays on user behavior. The results not only extend the application field of the first impression effect theory, but also have important guiding significance for the image management and personal branding of doctors on online medical platforms.

This study is organized as follows. Section 2 is the literature review and hypotheses. Section 3 presents the data and research methods. Section 4 presents the results of the empirical analysis. Section 5 discusses the findings in detail and gives suggestions and recommendations.

## 2 Literature

### 2.1 First impression effect

The study of the first impression has a long history, especially in the field of psychological research. The first impression effect is explained as an effect of “preconceptions” [8]. The first impression effect emphasizes the influence of the first input information through “first impression” on subsequent cognition in social cognition [25]. The first impression once formed, whether good or bad, will be dominant in others’ brains and last a long time. The first impression formed by both parties in the communication process will have a profound impact on the subsequent communication, judgment, and behavioral decision [8]. In particular, the first impression presented by facial features is always the first focus of attention [7,26]. Facial features are one of the most intuitive and rapid sources of information in social interactions. Facial features have long been an important factor in human decision-making processes. In the absence of other information, people often tend to evaluate other people’s personality traits and certain social behaviors based on facial features [7]. For example, the first impressions presented by facial features can lead to judgments about the competence, reliability, and trustworthiness of others [26]. A large number of empirical studies have found that facial features can affect people’s judgment of personal trust, leadership ability, and personality.

However, despite the abundance of research on first impression, fewer studies have applied this theory to online medical services. There is still a lack of comprehensive understanding of how facial features in physician images specifically affect user behavior and decision-making in the context of online medical platforms. This approach represents a novel application of first impression effect theory in online healthcare. Our goal is to explore the role of the physician’s facial features in shaping the patient’s initial perception and subsequent medical decisions. By focusing on this underexplored intersection, we hope to provide new insights into the complex dynamics of online doctor-patient interactions.

In the medical field, the emergence of online medical platforms not only provides users with convenient and quick medical services, but also has a profound impact on users’ medical treatment behavior [1]. In particular, the doctor’s online presence plays a key role in user decision-making. Because users often first get to know doctors through online profile photos [4]. In recent years, many scholars have conducted in-depth research on this issue. Studies have shown that photos of doctors and their professional backgrounds on online medical platforms can significantly improve users’ trust and satisfaction [12]. The doctor’s facial features and body language can also play an important role in first impression formation [11]. Tates et al. also found that doctors’ facial features, such as smiles and eye contact, can enhance users’ affinity perception, thus increasing users’ willingness to choose offline medical services [27]. Online healthcare platforms are realizing this critical point. Many platforms encourage doctors to post clear and professional photos to attract users better [4]. When the doctor’s online image is consistent with what users expect, users are more likely to choose offline medical services provided by the doctor. However, it has also been argued that over-reliance on first impressions may lead users to make irrational decisions [19]. Users sometimes overlook the doctor’s real medical ability and experience because of his or her appearance and background. Therefore, users still need to fully evaluate the doctor’s practical competence and reputation to make an informed decision.

### 2.2 The direct effect of online doctor image on user behavior

In today’s digital world, images play a crucial role in shaping perceptions and decisions. For instance, U.S. presidential candidates use personal photos on Instagram to build public rapport, where strong visual engagement can positively influence voter opinions, even for unfamiliar candidates [28]. Similarly, in e-commerce, user-generated review images enhance trust and mental imagery more effectively than text, impacting purchasing choices [29]. Additionally, societal biases like the “beauty premium” persist, where attractive individuals are often perceived as more competent and prosocial, reinforcing the “pretty is good” stereotype. These dynamics highlight how visuals drive engagement, trust, and judgment across social and commercial contexts [30,31].

In online medical platforms, users’ aesthetic preferences for doctor images can significantly shape decision-making and relationship-building. Studies demonstrate that doctors perceived as physically attractive enhance user satisfaction and treatment compliance, primarily by fostering trust and perceived competence [32]. Additionally, physical attractiveness is often interpreted as a proxy for communication skills, a key factor in patient-doctor interactions [4]. Visually appealing doctor profiles serve as heuristic cues, enabling users to infer professionalism and interpersonal effectiveness, which increases both online engagement and offline consultation bookings. As analyzed above, we propose the following hypothesis:

**H1: The appearance attractiveness of doctor images will positively impact users’ offline conversion rate.**

Smiling is a facial expression that fosters positive responses, enhancing perceptions of likability and friendliness [34]. In marketing, smiling is seen as a powerful tool, which not only triggers consumer trust and satisfaction through emotional contagion, but also boosts brand appeal [20]. Research has shown that smiling salespeople are more likely to engage consumers and prompt them to buy products [33]. The smile of an advertising spokesperson will increase customers’ emotional identification with the brand and enhance their brand loyalty. In the online medical environment, face-to-face communication between users and doctors is lacking. Therefore, users are more likely to evaluate doctors based on the information provided by their images [12]. Studies have shown that in doctor-user interaction, smiling can not only shorten the communication distance and relieve users’ tension, but also improve users’ perceived trust [34]. Users are likely to be influenced by the smiles in the doctor’s images and choose to consult doctors with higher levels of smiles both online and offline. Based on the above analysis, we propose the second hypothesis:

**H2: The expression of smiles in doctor images will positively impact users’ offline conversion rate.**

Gender difference is a complex and sensitive topic that is widely discussed in all sectors of society. Although gender equality has made great progress in the corporate world, men are still considered to have more authority and credibility in certain special positions [35]. Research shows that even with the same qualifications and abilities, men still have an advantage in obtaining senior positions and leadership roles [35]. In some intellectual and technical jobs, men are more likely to be hired [36]. Although more and more women are making their mark in STEM (science, technology, engineering and mathematics) fields, the proportion of male workers is still predominant. Globally, a UNESCO report shows that among STEM researchers worldwide, women account for only 29.3%, while men make up approximately 70.7%.

In the medical field, male doctors tend to be more popular and competent in the medical field due to their superior performance [37]. Studies have found that in male-dominated specialties (e.g., surgery, radiology), referral rates and procedural volumes tend to be higher [38]. And male physician were consistently paid 6% more than female physician [38]. Some patients believe that male doctors are more authoritative in “technical specialties” (such as surgery), while female doctors are more often associated with “caring specialties” (such as general practice), affecting the distribution of occupations [39]. Based on the above analysis, we infer that male doctors will positively affect their consultation behavior. Therefore, we assume that:

**H3a: Male doctors’ images will positively impact users’ offline conversion rate.**

However, there are exceptions to the “men are more capable” stereotypes. For example, in general surgery, there is no significant difference in surgical results between male and female physicians [40]. In general surgery, compared with male doctors, female doctors do not provide excessive medical resources and have a lower mortality rate [41]. Studies have also found that female users were more likely to regard female physicians as their preferred medical service providers [42]. Patients often seek not only medical expertise but also emotional support during the healthcare journey [42]. Therefore, it is reasonable to speculate that female doctors positively affect the offline conversion rate of users. Based on this, combined with H3a, we propose that:

**H3b: Female doctors’ images will positively impact users’ offline conversion rate.**

### 2.3 The moderating effect of efficacy satisfaction evaluation

User satisfaction evaluation is an important index to measure the quality of medical services [43]. Especially on online medical platforms, users are unable to interact directly with doctors, and therefore rely more on other users’ evaluations and recommendations [44]. The physician’s electronic word of mouth plays an important role in user decision-making. On the online medical platform haodf.com, the doctor’s satisfaction evaluation can be seen in the user reviews section of his or her homepage.

In our study, efficacy satisfaction evaluation is a direct evaluation of physician expertise by users. As an evaluation of doctors’ skills, efficacy satisfaction evaluation has a significant impact on users’ choice of doctors, because doctors with professional skills are more likely to be trusted and selected [13]. In addition, reputation evaluation may be more important when the two factors of doctor image and doctor reputation appear simultaneously [18]. A doctor with a good reputation will be more easily chosen by users, even if he or she is relatively less attractive. Based on this, we suggest that a doctor’s efficacy satisfaction evaluation may weaken the positive effect of doctors’ appearance attractiveness on users’ offline conversion rate. Therefore, the following hypothesis is proposed:

**H4a: Efficacy satisfaction evaluation significantly moderates the effect of appearance attractiveness on users’ offline conversion rate. Moreover, efficacy satisfaction evaluation will weaken the relationship between appearance attractiveness and users’ offline conversion rate.**

People tend to make preliminary observations and judgments based on facial expressions [20]. For example, in the eyes of users, smiling makes doctors more attractive [36]. However, when doctors produce a halo effect because of their superior ability, the initial advantages of the smile expression may be overlooked and the impact on the user may be mitigated and diminished. Based on this, we believe that users may pay less attention to the doctor’s smile expression when the doctor’s professional skills are evaluated better. Therefore, we propose the hypothesis:

**H4b: Efficacy satisfaction evaluation significantly moderates the effect of the smile on users’ offline conversion rate. Moreover, efficacy satisfaction evaluation will weaken the relationship between the smile and users’ offline conversion rate.**

Due to the influence of sociocultural and gender roles, men may be considered more authoritative and professional in some professional fields [35]. However, gender is not the only concern when it comes to dealing with practical issues. For example, in the actual consultation process, users value the experience and ability of the doctor more than gender. Based on this, we conclude that efficacy satisfaction evaluation may negatively regulate the relationship between male physicians and users’ offline conversion rate. Therefore, we propose the hypothesis:

**H4c: Efficacy satisfaction evaluation significantly moderates the effect of gender on users’ offline conversion rate. Moreover, the higher the efficacy satisfaction evaluation, the weaker the relationship between male doctors and users’ offline conversion rate.**

### 2.4 The moderating effect of attitude satisfaction evaluation

Attitude is one of the main factors in predicting an individual’s behavioral intention. In the field of consumer behavior, attitude has a direct impact on purchase intention and purchase behavior [45]. When consumers have a positive attitude toward a brand or product, they are more likely to choose to buy it. On the Internet medical platform, the attitude satisfaction evaluation can be regarded as the user’s direct evaluation of the doctor’s attitude and behavior in the service process, mainly including communication skills, politeness, humanistic care, and so on [46]. Good doctor-user communication can help users more fully understand the risk of disease occurrence.

When synchronously considering the basic characteristics of the doctor (such as appearance, age, gender, etc.) and the performance of actual contact, the influence of the doctor’s initial impression on the user’s decision may be weakened [19]. Studies have shown that most users think that the doctor’s appearance is important, but not as important as empathy and politeness [47]. Based on this, we expect that attitude satisfaction evaluation will weaken the effect of appearance attractiveness on users’ offline conversion rate. Therefore, we propose the hypothesis:

**H5a: Attitude satisfaction evaluation significantly moderates the effect of appearance attractiveness on users’ offline conversion rate. Moreover, efficacy satisfaction evaluation will weaken the relationship between appearance attractiveness and users’ offline conversion rate.**

The influence of eWOM on users’ decision-making has extended to the special service area of healthcare. Similar to online shopping, consumers tend to browse user reviews before making a purchase [45]. This phenomenon also exists in the Internet medical community. Users measure the quality of doctors’ services through online service evaluations [46]. As a result, even doctors with amiable faces may not be selected by users if they have a bad reputation online, especially with a bad attitude during treatment. Based on this, we suggest that attitude satisfaction evaluation may weaken the relationship between smiling and users’ offline conversion rates. Therefore, we propose:

**H5b: Attitude satisfaction evaluation significantly moderates the effect of the smile on users’ offline conversion rate. Moreover, efficacy satisfaction evaluation will weaken the relationship between the smile and users’ offline conversion rate.**

The doctor’s attitude can have a profound effect on a user’s behavior. For example, a doctor seen as polite and user may be more likely to be trusted and chosen by users, regardless of gender. Based on this, we infer that high scores for physician attitudes may weaken the relationship between gender and users’ offline conversion rate. Therefore, the hypothesis is proposed:

**H5c: Attitude satisfaction evaluation significantly moderates the effect of gender on users’ offline conversion rate. Moreover, the higher the attitude satisfaction evaluation, the weaker the relationship between male doctors and users’ offline conversion rate.**

**Fig 1:**
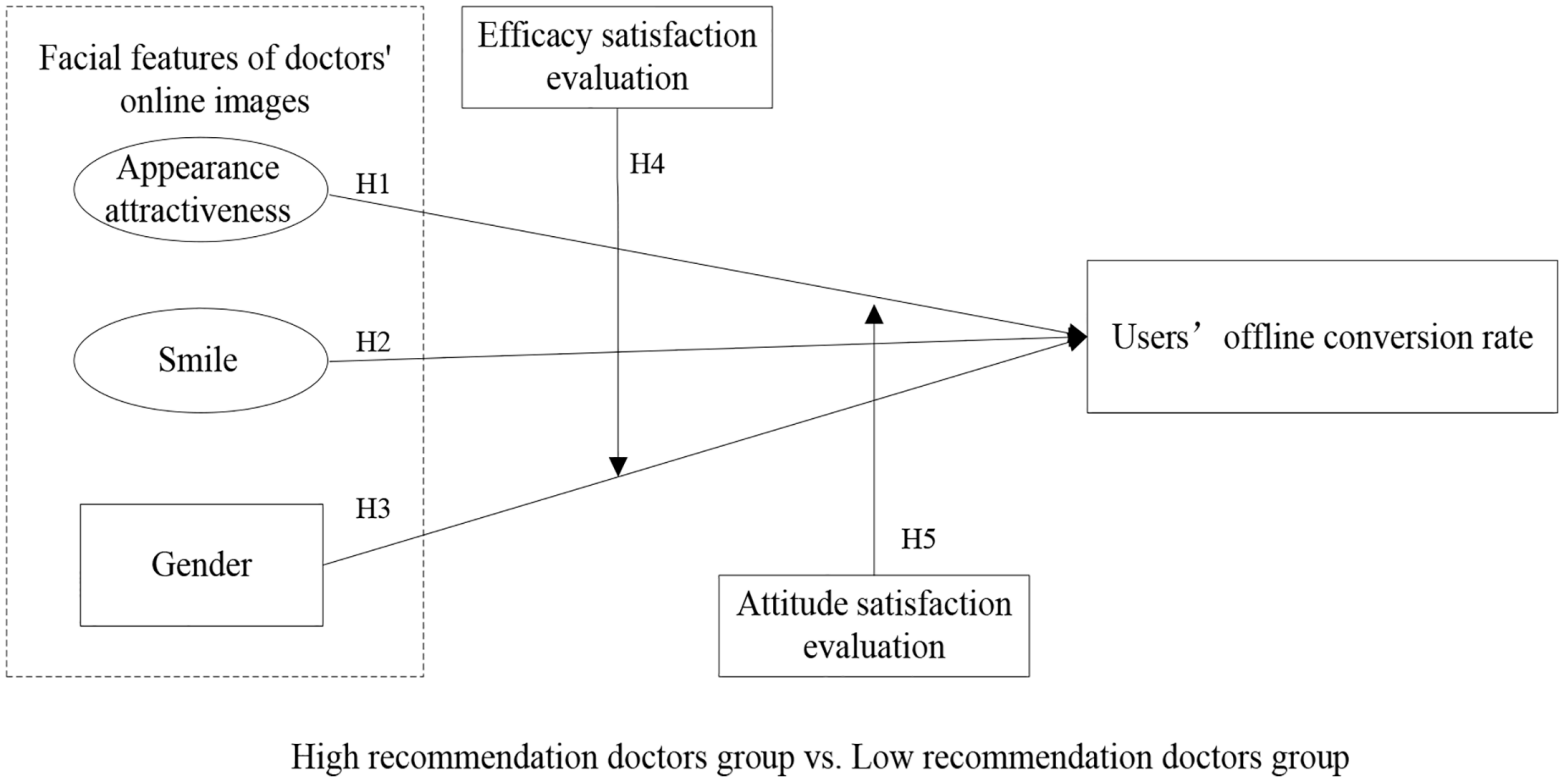
Theoretical model‌‌.

A theoretical model is proposed in this study, as shown in Fig 1. The model explains how facial features of physician images affect users’ offline conversion rate, as well as the moderating effects of efficacy and attitude satisfaction evaluation. In addition, group regression was conducted according to the recommendation degree of the doctor’s homepage to explore the reasons affecting the heterogeneity of users’ decision-making.

## 3 Methodology

### 3.1 Data acquisition and processing

The data used in this study were obtained from the leading online healthcare platform “Haodf” in China (https://www.haodf.com/). It comprehensively covers a variety of medical specialties and services, and provides one-stop solutions to medical problems for online services and offline treatment [12]. The reason why we chose “haodf” is that it has a comprehensive database of doctors, including various types of data such as images, evaluations, and the number of visitors [48,49]. The diversity and uniqueness of the data provide a good basis for our research. This confirms that the data collection and analysis methods comply with the terms and conditions of haodf.

**Fig 2:**
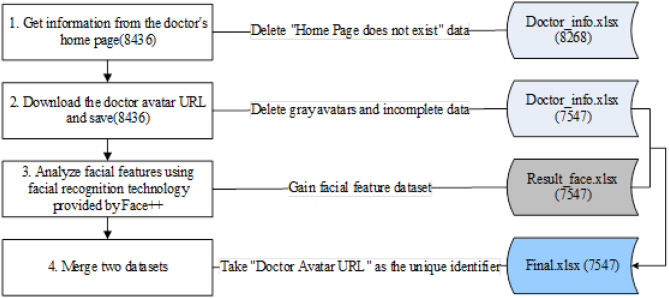
Data processing procedure.

We used Python to collect physician data from four major departments (including Internal Medicine, Surgery, Obstetrics & Gynecology, and Pediatrics) on haodf.com. A stratified random sampling strategy was employed, with systematic sampling (fixed interval) applied within each department layer based on the platform’s default sorting. The final dataset consists of 8,436 unique physician profiles, each representing a complete record. After deleting incomplete avatars and information, we finally obtained 7,547 valid pieces of data. Then, we used the Face++ algorithmic for facial recognition and analysis. The Face++ algorithmic engine is implemented by the Chinese company Megvii, which provides a series of vision technology solutions based on complex and advanced technologies [14]. Face++ algorithmic is widely used in the research of social media, sharing economy, and online images, which has been proven to have high accuracy and reliability [21,22,50,51]. Face++ achieves an accuracy rate of up to 97% in face recognition (including face detection, key point alignment, and feature extraction). Face++ uses a face detection algorithm to locate the face in the image, then aligns the face to standardize its angle and position. Finally, it extracts facial features like contours, eyes, nose, and mouth, capturing their position, shape, and texture [22]. For example, the Beauty scoring system uses AI to examine your face and boils down its results into a percentage grade of likely attractiveness [52]. Fig 2 shows the data acquisition and cleaning process.

### 3.2 Research variables

The dependent variable used in this study is users’ offline conversion rate (Uocr). It is measured by the ratio of reported users to total users (%) as shown on the haodf.com platform. The datasets “reported users” and “total users” are both obtained from the doctor’s homepage. Offline conversion rate usually represents the percentage of offline actions that occur after the online activity [53]. In this study, “users’ offline conversion rate” refers to the percentage of users who actually choose to visit a hospital or clinic for face-to-face consultation and treatment with the doctor after browsing, initially understanding, and consulting the doctor on an online medical platform. As a dependent variable, it represents the convergence effect and conversion efficiency of online and offline medical services. It reflects the effectiveness of the online medical platform and the actual demand for medical services. A high offline conversion rate indicates that users have great trust in health information, doctor image, and consulting services displayed on the online platform [54], and are willing to take this as a basis for the actual offline medical treatment action.

In regression analysis, we use logarithmic transformations to preprocess the dependent variables. In statistics, a logarithmic transformation is taking the logarithm of a variable. Because Uocr is proportional data, which is highly dispersed. Using logarithmic conversion not only helps to stabilize the variance and linearization relationship, but also helps to reduce the data volatility and make the data closer to the normal distribution. After taking the logarithm, the coefficient can be interpreted as a relative change in the original ratio, which is consistent with the actual meaning of the original data.

*Independent variables.* The independent variables in our study are the doctor’s appearance attractiveness, smile, and gender. These are the main facial cues included in a doctor’s online profile picture. To operationalize the abstract concept of “appearance attractiveness (Apattr),” we evaluated doctors’ facial attractiveness using a composite score derived from three dimensions: (1) symmetry (e.g., facial balance and proportionality), (2) averageness (deviation from population-average facial features), and (3) expressiveness (e.g., warmth and approachability inferred from expressions in profile photos). These metrics were quantified through a combination of machine learning-based facial analysis tools and ratings from independent human evaluators to ensure robustness. Face++ evaluates the appearance attractiveness of an image from the perspective of male and female respectively, and gives the male_score and the female_score. These two scores reflect the subjective rating of appearance attractiveness in the images by gender. Finally, in order to overcome the gender bias of attractiveness, the two scores are averaged to obtain a composite score representing the image’s Apattr variable. For the smile variable, the API returns a floating-point number ranging from 0 to 100 based on the analysis results, with higher values indicating higher smile levels. For gender, the API returns string data: male and female. For the convenience of subsequent regression analysis, we set gender as a dummy variable (male = 1, female = 0). Dummy variables are used to represent attributes of categorical variables. The coefficient of the gender dummy variable represents the effect of male on the dependent variable compared to the reference category (female).

*Moderating variables.* Our study’s moderating variable is the user’s satisfaction evaluation with doctors’ post-diagnosis evaluation, including efficacy satisfaction evaluation (ESE) and attitude satisfaction evaluation [55]. Satisfaction represents the user’s expectations and the outcomes for access to healthcare services. User satisfaction evaluation with online consultation is an important factor in evaluating the comprehensive level of doctors, which can well evaluate the quality of medical services and doctor-user interaction [56].

Efficacy satisfaction evaluation is the user’s satisfaction with the treatment effect, including the improvement of symptoms, disease control and cure, focusing on the actual treatment results. Attitude satisfaction evaluation refers to the user’s satisfaction with the attitude and behavior of medical service providers, including the professional quality, communication skills, politeness, and care of doctors, focusing on the process of medical service and the service quality of medical personnel [46]. Both efficacy and attitude satisfaction evaluation are obtained from “evaluation” pages of online physicians.

*Controlling variable.* This research used “Total visit (T-visit)” as the control variable. The data is acquired from the online platform haodf.com and represents the total number of visits to a doctor’s homepage. The meanings of each variable are explained in Table 1.

**Table 1:** Variable interpretation.

	Variables	Variable definition
Dependent variable	Uocr	Users’ offline conversion rate (%). It is expressed by the ratio of reported users to total users.
Independent variable	Apattr	Appearance attractiveness. It represents the facial attractiveness of online doctor avatars.
	Smile	It represents the smiling degree of online doctor avatars.
	Gender	It indicates the gender of online doctor avatars.
Moderating variables	ESE	Efficacy satisfaction evaluation. It indicates the user’s satisfaction with the treatment effect.
	ASE	Attitude satisfaction evaluation. It indicates the user’s satisfaction with the service attitude and behavior.
Controlling variable	T-visit	Total visit. It represents the total number of visits to the doctor’s home page by the user.

### 3.3 Estimation strategy

Our empirical study used a linear regression model because our research question focused on the relationship between a continuous dependent variable (users’ offline conversion rate) and a series of independent variables (including online facial images of doctors and other control variables). Linear regression models can quantify this relationship and reveal the extent to which each independent variable influences the dependent variable through coefficient estimation.

To test our hypotheses of H1, H2, and H3a, we constructed the following model for the direct effect of the physician’s online facial images on users’ offline conversation rate:

$Uocr=\beta_{\text{0}}+\beta_{\text{1}}\times Apattr+\beta_{\text{2}}\times Smile+\beta_{\text{3}}\times Gender+\beta_{\text{4}}\times Control+\varepsilon$

where $\beta_{0}$ was the intercept term; $\beta_{1}$ to $\beta_{3}$ were coefficients of interested variables, and “Control” was the control variable. “ε” was the error term. Gender is a dummy variable. By introducing the gender dummy variable, we can directly estimate the effect of gender on the dependent variable.

To test the moderating effect of Efficacy satisfaction evaluation (ESE) in hypotheses H4a-H4c, we used the following model:


$$\begin{array}{cc} Uocr=\; & \beta_{\text{0}}+\beta_{\text{1}}\times Apattr+\beta_{\text{2}}\times Smile+\beta_{\text{3}}\times Gender+\beta_{\text{4}}\times ESE\times Apattr+\beta_{\text{5}}\times ESE\times Smile\\ & \;\;+\beta_{\text{6}}\times ESE\times Gender+\beta_{\text{7}}\times Control+\varepsilon \end{array}$$

Where $\beta_{4}$ to $\beta_{6}$ were coefficients of interested variables.

To test the moderating effect of Attitude satisfaction evaluation [55] in hypotheses H5a-H5c, we used the following model:


$$\begin{array}{cc} Uocr=\; & \beta_{\text{0}}+\beta_{\text{1}}\times Apattr+\beta_{\text{2}}\times Smile+\beta_{\text{3}}\times Gender+\beta_{\text{4}}\times ASE\times Apattr+\beta_{\text{5}}\times ASE\times Smile\\ & \;\;+\beta_{\text{6}}\times ASE\times Gender+\beta_{\text{7}}\times Control+\varepsilon \end{array}$$

Where $\beta_{4}$ to $\beta_{6}$ were coefficients of interested variables.

This study used statistical analysis software – Stata- to analyze the obtained data and conduct empirical research.

## 4 Results

### 4.1 Hypotheses testing

Table 2 presents the descriptive statistics for all variables included in this study. The sample consists of 7,547 observations. The dependent variable, lnUocr, has a mean of 0.811 and median of 0.552, suggesting a right-skewed distribution. Both ESE and ASE show means near 1 with very low standard deviations. In this study, ESE and ASE are the moderating variables, and we centre the variables for vif tests.

**Table 2:** Descriptive statistics.

Variables	N	Mean	Std. Dev.	Min	Max	Median
lnUocr	7,547	0.811	0.838	0	7.215	0.552
ESE	7,547	0.989	0.036	0.4	1	1
ASE	7,547	0.991	0.033	0.4	1	1
Apattr	7,547	0.517	0.148	0	1	0.517
Smile	7,547	0.516	0.445	0	1	0.079
Gender	7,547	0.702	0.458	0	1	1
T-visit	7,547	0.500	0.453	0.521	1.995	0.543

Apattr displays a roughly symmetric distribution with moderate spread. Meanwhile, to eliminate the effect of magnitude and to address the problem of multicollinearity, we normalised the Smile and Apattr variables. Gender is a binary variable with a mean of 0.702, indicating that approximately 70.2% of the sample falls into the category coded as 1. Finally, since the data range of T-visit is significantly large, we standardized T-visit to align its scale with other variables. This standardization process ensures consistent scaling while preserving the original distribution shape of the data.

The severity of multicollinearity between variables can be measured by the VIF test. The larger the VIF value, the stronger the correlation between the variable and the other independent variables [57]. In this study, the VIF value of Apattr is 1.04, that of Smile is 1.16, and that of Gender is 1.17. The VIF value of ESE is 2.10, and that of ASE is 2.23. VIF test results are all less than 5, indicating that there is no multicollinearity problem among variables [57].

**Fig 3:**
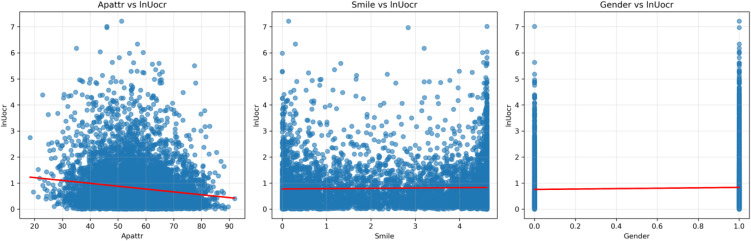
Linearity Check.

Fig 3 shows three sets of scatter plots examining the relationship between each of the three independent variables and the dependent variable. From the trend lines, linearity is largely satisfied.

**Fig 4:**
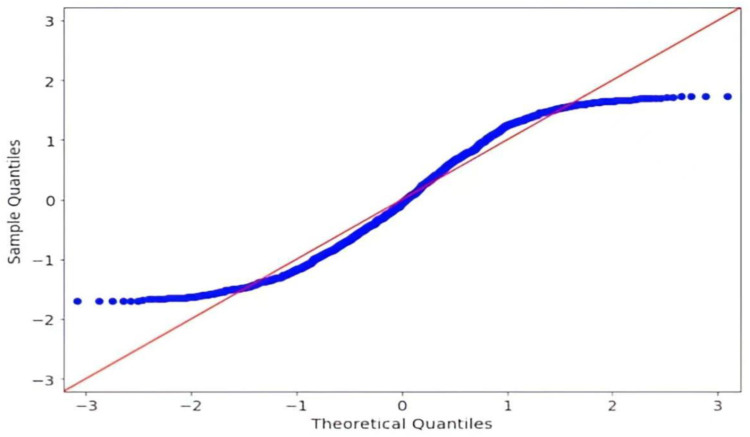
Residual Normality Test.

Fig 4 shows the results of the residual normality test. The points shown in the figure are roughly distributed along the diagonal, which indicates that the sample data follow a roughly normal distribution.

**Fig 5:**
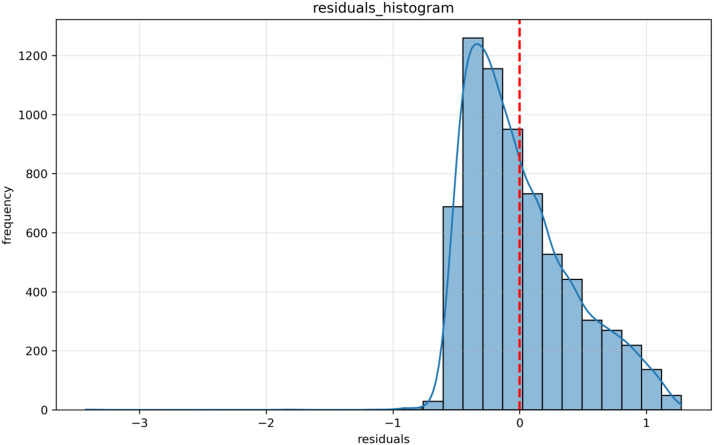
Histogram of Residuals.

Fig 5 shows the histogram of residuals, which represents the difference between the observed values in the regression model and the predicted values in the model. The distribution of residuals shows a “bell-shaped” trend, which is basically consistent with the assumption of error normality in linear regression. Most of the residuals are concentrated around 0, indicating that the model predictions are more accurate.

### 4.2 Hypotheses testing

The empirical results are shown in Table 3. Model 1 performed regression analysis of the control variable and independent variables associated with the physician’s online facial features. The relationship between each variable and the dependent variable can be obtained from the second column of Table 3. First, H1 is supported. Because there is a significant positive association (β = 0.006, p < 0.001) between the physician’s online appearance attractiveness and users’ offline conversion rate. Second, the doctor’s smile is positively correlated with users’ offline conversion rate and is significant (β = 0.002, p < 0.001). So H2 is supported. Third, the physician’s gender is positively correlated and significant with users’ offline conversion rate (β = 0.223, p < 0.001). Because in this study, we set the male doctor to 1. So, the results explain male doctors have a significant positive impact on users’ offline conversion rate. So, H3a is also supported. The choice of “T-visit” (Total Visit) as a control variable was made to exclude confounding bias. The regression results demonstrated that this variable exhibited strong predictive power for the dependent variable (p < 0.001), albeit with a relatively small coefficient value. This is attributed to the variable retaining its original dimension, where the marginal effect represents the minute change induced by each additional visit.

**Table 3:** Regression results.

DV: lnUocr	Model 1	Model 2	Model 3
Apattr	0.006*** (15.08)	0.001 (0.09)	0.002 (0.17)
Smile	0.002*** (12.00)	0.009* (1.66)	0.008 (1.37)
Gender (Male)	0.223*** (10.37)	1.161** (2.03)	1.878*** (2.79)
ESE		1.241*** (23.11)	
ASE			1.229*** (22.96)
ESE *Apattr		−0.013 (−1.15)	
ESE *Smile		−0.009 (−1.58)	
ESE *Gender (Male)		−1.067* (−1.85)	
ASE *Apattr			−0.014 (−1.03)
ASE *Smile			−0.008 (−1.30)
ASE *Gender (Male)			−1.788*** (−2.63)
T-visit	0.000*** (25.06)	0.000*** (22.05)	0.000*** (22.04)
N	7,547	7,547	7,547
R^2^	0.436	0.471	0.472
R^2^_a	0.435	0.471	0.472
F	1586	915.4	918.7

**Note(s):** *p < 0.05, **p < 0.01, ***p < 0.001.

Model 2 adds the moderating effect of efficacy satisfaction evaluation based on model 1. Column 3 of Table 3 shows the interaction coefficients of efficacy satisfaction evaluation (ESE) with independent variables. First, the main effect of Apattr is positively correlated but not significant in the moderating effect of ESE (β = 0.001, p > 0.05). The interaction coefficient of ESE *Apattr is negative but not significant (β = −0.013, p > 0.05). Therefore, ESE does not significantly regulate the effect of appearance attractiveness on users’ offline conversion rate. H4a is rejected. Second, the main effect of Smile is positive and significant in the moderating effect of ESE (β = 0.009, p < 0.05). The interaction coefficient of ESE *Smile is negative but not significant (β = −0.009, p > 0.05). Therefore, ESE does not significantly regulate the effect of the smile on users’ offline conversion rate. H4b is also rejected. Third, the main effect of Gender is positive and significant in the moderating effect of ESE (β = 1.161, p < 0.01). The interaction coefficient of ESE *Gender is negative and significant (β = −1.067, p < 0.05). Therefore, ESE significantly moderates the effect of gender on users’ offline conversion rate, and the regulatory effect is negative. So H4c is supported.

Model 3 adds the moderating effect of attitude satisfaction evaluation based on model 1. Column 4 of Table 3 shows the interaction coefficients of attitude satisfaction evaluation [55] with independent variables. First, the main effect of Apattr is positively correlated but not significant in the moderating effect of As (β = 0.002, p > 0.05). The interaction coefficient of ASE *Apattr is negative but not significant (β = −0.014, p > 0.05). Therefore, ASE does not significantly regulate the effect of appearance attractiveness on users’ offline conversion rate. H5a is rejected. Second, the main effect of Smile is positive but not significant in the moderating effect of ASE (β = 0.008, p > 0.05). The interaction coefficient of ASE *Smile is negative but not significant (β = −0.008, p > 0.05). Therefore, ASE does not significantly regulate the effect of the smile on users’ offline conversion rate. H5b is also rejected. Third, the main effect of Gender is positive and significant in the moderating effect of ASE (β = 1.878, p < 0.001). The interaction coefficient of ASE *Gender is negative and significant (β = −1.788, p < 0.001). Therefore, ASE significantly moderates the effect of gender on users’ offline conversion rate, and the regulatory effect is negative. So H5c is supported.

To ensure the reliability and accuracy of the regression results, we conduct a robustness check on the model. The robustness check is usually done to verify that a result is consistent or stable under different settings. A robust model can handle outliers and ensure the stability of the parameters without large fluctuations in the results. The specific robustness check results are shown in Table 4.

**Table 4:** Robust check.

DV: lnUocr	Model 1	Model 2	Model 3
Apattr	0.006*** (15.93)	0.001 (0.09)	0.002 (0.14)
Smile	0.002*** (11.29)	0.009 (1.29)	0.008 (0.96)
Gender (Male)	0.223*** (10.17)	1.161* (1.86)	1.878** (2.45)
ESE		1.241*** (20.68)	
ASE			1.229*** (20.56)
ESE *Apattr		−0.013 (−1.10)	
ESE *Smile		−0.009 (−1.23)	
ESE *Gender (Male)		−1.067* (−1.69)	
ASE *Apattr			−0.014 (−0.84)
ASE *Smile			−0.008 (−0.91)
ASE *Gender (Male)			−1.788** (−2.32)
T-visit	0.000*** (6.64)	0.000*** (6.68)	0.000*** (6.70)
N	7,547	7,547	7,547
R^2^	0.436	0.471	0.472
R^2^_a	0.435	0.471	0.472
F	1477	820.3	821.2

**Note(s):** *p < 0.05, **p < 0.01, ***p < 0.001.

### 4.3 Heterogeneous effect

This study examined the heterogeneous effects of appearance attractiveness, smile, and gender among online doctors with different “user recommendation degree”. “User recommendation degree” is the recommendation index of users to doctors, which comes from the information of the doctor’s homepage on the haodf.com platform [58]. It is distinguished from “user satisfaction evaluation”. It reflects the user’s willingness to recommend this doctor to others and generally occurs after the satisfaction evaluation.

Table 5 shows the heterogeneous results. Overall, the doctor’s appearance attractiveness, smile, and gender have significant positive effects on users’ offline conversion rate regardless of whether they are high-recommended or low-recommended. Firstly, it can be seen from the table that the beauty premium effect of highly recommended doctors is more significant. For each unit increase in attractiveness score, users’ offline conversion rate of low and high referral physicians increased by 0.5% and 0.6%, respectively. However, by the coefficient equality test [59], there is no statistical significance between the two groups (Z = 1.314, p > 0.05). Secondly, the smile effect of low-recommended doctors is more significant. For each unit increase in smile score, users’ offline conversion rate of low and high referral physicians increased by 0.4% and 0.1%, respectively. The coefficient equality test shows that smiling is significantly enhanced in low-recommended physicians (Z = 7.307, p < 0.001). Finally, the gender effect of doctors with low recommendations is more significant. For every one-unit increase in gender score, users’ offline conversion rate of low and high referral physicians increased by 31.2% and 16.4%, respectively. The test for the equality of coefficients implies that gender is significantly enhanced among low-recommended physicians (Z = 3.481, p < 0.001).

**Table 5:** Determinants of users’ offline conversion rate by recommendation.

	(1) lnUocr	(2) lnUocr
	Low recommendation	High recommendation
Apattr	0.005***	0.006***
	(0.0006)	(0.0005)
Smile	0.004***	0.001***
	(0.0003)	(0.0002)
Gender	0.312***	0.164***
	(0.0335)	(0.0261)
Obs.	3,033	4,514
R-squared	0.414	0.491
r2_a	0.413	0.491
F	733.2	980.2

**Note(s):** *p < 0.05, **p < 0.01, ***p < 0.001.

## 5 Discussion and conclusion

### 5.1 Research conclusions

This part discusses the mechanism by which the doctor’s facial features affect users’ offline conversion rate, and the moderating effect of satisfaction evaluation as eWOM. The explanation mechanism is as follows:

First of all, we explain the mechanism of influence of appearance attractiveness, smile, and gender (male) on the main effect of users’ offline conversion rate.

I *Online doctors’ appearance attractiveness has a positive effect on users’ offline conversion rate.* This suggests that as doctors’ appearance attractiveness improves, users’ offline conversion rate is increasing. Existing studies have shown that people tend to hold more positive evaluations of physically attractive people and are more inclined to interact with them [30]. In the online medical environment, doctors with high appearance attractiveness may make a good first impression on users. They tend to be seen as more professional and knowledgeable, able to provide more satisfying services and higher medical value [30,32], thus triggering the emotional trust of users and enhancing their intention to choose the doctor offline. However, our results are contrary to the previous findings of Ouyang and Wang [4]. This may be due to the differences in sample selection scope and data collection methods. Our results also bring the “beauty premium” phenomenon commonly seen in marketing to the realm of online medical services.
II *Smiling doctors have a positive effect on users’ offline conversion rate.* This shows that the higher the smile degree of doctors, the higher users’ offline conversion rate. This finding not only validates the previous theory that smiling is generally associated with friendliness, trust, and positive emotions [60], but also provides new insights into the field of online medical advice. The doctor’s smile can provide nonverbal positive feedback. When doctors wear a smile, it reduces the user’s anxiety and tension, and makes it easier to build good relationships and trust with users [34,36]. These positive effects will facilitate the decision-making process of users from online consultation to offline consultation.
III *Male doctors have a positive effect on users’ offline conversion rate.* This means that male doctors may be more likely to promote users’ offline conversion rate than female doctors. This phenomenon can be caused by serious factors such as cultural and social factors, users’ gender preferences, and traditional gender stereotypes. On the one hand, different cultures have different perceptions of professional authority. For example, some cultures place more emphasis on patient-centricity. In such a culture, female doctors may stand out for their empathy and communication skills [61]. Conversely, male doctors may be preferred in cultures that value assertiveness and authority [35]. On the other hand, users may prefer to communicate and interact with male physicians who have a more direct and authoritative style [62]. Although both sexes perform well in the medical field, the traditional gender stereotype may cause users to trust and prefer male doctors more and choose them for offline consultations.

Second, we explain the mechanism of effect under the moderation of efficacy satisfaction evaluation.

I *The main effect of doctors’ appearance attractiveness is positive but not significant under the moderating of efficacy satisfaction evaluation.* This means that when considering the professional competence and treatment effect of doctors, the influence of the doctor’s appearance on users’ offline consultation behavior is not strong [18]. Users will no longer pay attention to the superficial characteristics of the doctor. Efficacy satisfaction evaluation has no significant moderating effect on the relationship between the doctor’s appearance attractiveness and users’ offline conversion rate*.* The reasons for the insignificant adjustment effect were further analyzed. Considering the weight of doctors’ professional competence and treatment effect in users’ minds, in some cases, the health status of users and the treatment effect of doctors may be their most concerned issues. Therefore, when users are very satisfied with the results of treatment, the physician’s appearance may become less important in the user’s decision to continue treatment [63]. This also re-emphasizes the core position of medical quality and efficacy satisfaction evaluation in the doctor-user relationship.
II *Under the moderating effect of efficacy satisfaction evaluation, the main effect of the smile is positive and significant.* Efficacy satisfaction evaluation has no significant moderating effect on the relationship between the doctor’s smile and users’ offline conversion rate. This indicates that there was no significant change in the relationship between smiling and users’ offline conversion rates regardless of efficacy satisfaction evaluation. The doctor with a smile may help push users to continue receiving services from the medical provider or institution. Previous research also supports the result. For example, non-verbal communication such as smiling can predict a user’s health status [64]. The way doctors interact with users (such as smiling) will affect the user’s trust and satisfaction [36].
III *Efficacy satisfaction evaluation significantly negatively moderates the effect of male doctors on users’ offline conversion rate.* This means that as efficacy satisfaction evaluation increases, the positive impact of male physicians on users’ offline conversion rate diminishes. This not only demonstrates the importance of word-of-mouth satisfaction evaluation in users’ choice of medical services [65], but also indicates the weakening of gender discrimination in the medical field. In other words, if a user is satisfied with the results of the treatment, he will reduce the consideration of the doctor’s gender when he continues the offline treatment.

Finally, we explain the mechanism of effect under the moderation of attitude satisfaction evaluation.

I *Under the moderating effect of attitude satisfaction evaluation, the main effect of the doctor’s appearance attractiveness and smile are positive but not significant.* Attitude satisfaction evaluation does not significantly regulate the effect of the doctor’s appearance attractiveness and smile on users’ offline conversion rate. This means that when doctors demonstrate a good service attitude, appearance and smile will no longer be the primary factors driving user decisions [47]. And, the doctor’s attitude satisfaction evaluation has little effect on the results.
II *Attitude satisfaction evaluation significantly negatively moderates the effect of male doctors on users’ offline conversion rate.* This indicates that when the doctor’s satisfaction with service attitude is higher, the positive impact of male doctors on users’ offline conversion rate decreases. This means that gender becomes less important when doctors (whether male or female) demonstrate a satisfactory attitude toward user care. This also explains, to some extent, the decrease in sexism in contemporary society, as well as the changing perception of the bias against men generally having more authority and ability [35].

Our research not only provides valuable market insights for the medical service industry, but also has a profound impact on the research field of eWOM and visual information processing on online platforms. In addition to traditional service quality and price factors, visual elements such as doctor images and smiles should also be taken into account by eWOM. These elements are easier to be perceived and remembered by users in the network environment, thus affecting users’ decision-making process. At the same time, online platforms should fully consider the role of visual elements when processing and displaying information. By optimizing the doctor image can significantly enhance the user’s trust and satisfaction, thus promoting users’ offline conversion.

Next, we interpret the results of heterogeneity analysis. Among highly recommended physicians, the doctor’s smile and gender have less impact on users’ offline conversion rate. This suggests that there may be differences in how users perceive physicians with high and low recommendations. As a result, users may have different expectations, needs, and decision-making mechanisms for doctors with different recommendation backgrounds [66]. For example, the highly recommended doctor’s professional reputation may already be established among users, so users may base their decisions more on the doctor’s professional advice. For low-recommended doctors, users may rely more on non-professional factors (such as smiling, gender, and other surface characteristics) to make judgments and decisions.

### 5.2 Theoretical contributions

Through empirical analysis, this study deepens people’s understanding of the correlation between the doctor’s facial images and users’ behaviors. The study has several theoretical contributions.

First, the findings highlight the impact of facial attributes as a key carrier of physician impression formation on the online platform. It is confirmed that the online image of the doctor is a key influencing factor for users’ decision-making. This paper reveals the possible mechanism of the impact of online doctor images on users’ offline conversion rate.

Second, the study expands the research field of the “beauty premium” phenomenon. The “beauty premium” phenomenon is initially demonstrated mainly in the labor market and consumer markets. This study extends it to the medical field. Through empirical analysis, it is found that the doctor’s appearance attractiveness has a significant positive impact on users’ offline conversion rate. This opens up a new area of research on the “beauty premium” phenomenon.

Third, the innovative research perspective of “first impression effect”. This study explores the judgment and choice behavior of users towards doctors from a psychological perspective. It not only contributes to a deeper understanding of how users choose their doctors, but also provides new ideas for improving the doctor-user relationship and users’ satisfaction.

### 5.3. Practical significance

This study explores the effect of the online doctor’s facial features on users’ offline conversion rate from the perspective of visual images, and the moderating effect of satisfaction evaluation as eWOM on this effect. These results provide references for users’ psychological mechanisms and behavioral decisions in choosing doctors. At the same time, it also provides practical significance for online medical platform management.

First, the platform should guide doctors to upload genuine and professional photos, avoiding excessive retouching or deliberately creating expressions (such as fake smiles). Ethical norms need to be established to ensure that photos are based on accuracy rather than artificially created attractiveness, thereby preventing patients from making biased judgments.

Second, while presenting the doctor’s photo, it is necessary to highlight their qualifications, patient evaluations and diagnosis and treatment data to reduce patients’ excessive reliance on facial features. The platform algorithm should avoid taking appearance alone as the basis for recommendation to ensure that all qualified doctors receive fair exposure.

Third, the platform should remind patients to pay attention to the unconscious biases that may exist when choosing doctors (such as judging people by their appearance). The platform can encourage users to comprehensively consider the qualifications and reputation of doctors through prompt messages or guiding words, rather than relying solely on visual features.

### 5.4. Limitations and future research

The study has several limitations. First, the source of the data. The data in this study are only from one domestic online medical platform “haodf.com”, which may not be a comprehensive representation of all online medical services. Future studies should consider integrating data from multiple online platforms in different cultural contexts to enhance the generality of the findings and reduce potential bias from the monocultural cultural and operational context of ‘haodf.com’. Second, the scope of variables is not limited to these three factors. In the future, other characteristics such as whether the doctor is wearing professional clothing, the angle of the head, or the photo environment could be included in the study to evaluate. Third, this study utilized the Face++ API to quantify the facial features of doctors. Future research might employ advanced image analysis techniques, such as Convolutional Neural Networks (CNN), to extract more nuanced facial features and potentially uncover deeper insights into how subtle aspects of a doctor’s image influence patient decisions. Additionally, further studies could explore variations across different medical specialties through hierarchical regression analysis, as this study focuses primarily on general patterns.
